# Inclusion of green fodder sorghum in the diet of lactating buffalo cows: influence on milk yield and composition, and economic sustainability

**DOI:** 10.3389/fvets.2026.1758156

**Published:** 2026-02-02

**Authors:** Francesco Capezzuto, Lorenzo Infascelli, Piera Iommelli, Francesco Franco, Federico Infascelli, Raffaella Tudisco

**Affiliations:** 1 Department of Veterinary Medicine and Animal Production, University of Napoli Federico II, Napoli, Italy; 2 Department of Economics and Law, University of Cassino and Southern Lazio, Cassino, Italy

**Keywords:** buffalo, fresh green sorghum, milk, nutrition, sustainability

## Abstract

**Introduction:**

Effects on production performance from dietary inclusion of green fodder Sorghum in lactating water buffalos were explored. Rising feed price volatility makes low-cost forage crops a strategic option to reduce dairy farming costs. The use of green fodder sorghum through the cut-and-carry system improves forage utilization, nutrient intake, and milk yield and composition.

**Methods:**

A total of 40 buffaloes, homogeneous in terms of milk yield, parity and body condition score (BCS), were selected and assigned to two groups: control group (C) and treated group (S). Experimental diets were offered to cover requirements as a Total Mixed Ration (TMR) with the inclusion of green fodder Sorghum (treated group, at an amount of 14.7% of TMR) and without (control group). The trial was conducted from July to September 2024. Milk samples were collected and analysed for chemical composition somatic cell count and urea. In addition, feed and faecal samples were collected to determine chemical composition and digestibility.

**Results:**

The inclusion of green fodder sorghum did not affect milk yield (C: 10.4 kg/head/d vs. S: 10.4 kg/head/d) or milk composition, with the exception of fat content, which was significantly higher in the treated group (C: 71.2 g/kg vs. S: 85.0 g/kg; *p* < 0.01). This increase was associated with improved digestibility of NDF and ADF of the experimental diet compared to the control diet (*p* < 0.01).

**Discussion:**

The economic assessment revealed that partially replacing corn silage and other dietary ingredients with green sorghum should reduce feeding costs by 0.27 EUR/head/day, making this strategy economically advantageous. Overall, the inclusion of green fodder sorghum proved beneficial in terms of cost efficiency and productive performance, aligning with the sustainability goals of the 2030 Agenda, particularly SDGs 12 and 13.

## 1 Introduction

Buffalo (*Bubalus bubalis*) farming represents a sector of considerable economic importance in southern Italy, primarily due to its role in the production of mozzarella cheese (1, 2) and, to a lesser extent, for meat (3). Although buffaloes digest fibrous feeds more efficiently than cattle (4), high energy and protein diets are formulated to increase milk yield, with high risk for negative outcomes on ruminal fermentations (5) as undesirable effect. Corn silage is widely used in the diets of lactating buffalo cows, despite requiring intense irrigation, raising concerns from economic and environmental sustainability perspectives (6). In this context, the interest in forages with less environment impact than corn is progressively increasing; particularly, sorghum is attracting attention given the possibility to be used, as forage or grain, especially as replacement of corn when agronomic practices must face water scarcity for irrigation. Indeed, the capacity of sorghum to tolerate periods of water scarcity and to restore vegetative activity when subjected to stress has been demonstrated (7). The partial or complete replacement of corn with alternative forages such as sorghum can also be seen as part of a broader strategy for the environmental, social and economic sustainability of buffalo farming, in line with the Sustainable Development Goals (SDGs) of the United Nations’ 2030 Agenda (8). Specifically, thanks to sorghum adaptability to adverse environmental conditions and its remarkable water use efficiency, it is possible to contribute concretely to achieving SDG 12 (Responsible Consumption and Production) and SDG 13 (Climate Action), significantly reducing water consumption and the ecological footprint of livestock farms. Compared with maize it needs lower inputs of water and nitrogen fertilizer (9, 10). The high-water efficiency of sorghum therefore allows for reduced use of irrigation and energy for water supply, with a positive environmental and economic impact. Moreover, in the current context, marked by increasing price volatility of feed and raw materials, the use of forage crops with low cultivation costs can be one of the strategic levers to contain feed expenses, which represent one of the main cost items in dairy farming. Green fodder sorghum in livestock farming is used by means of the “cut and carry” technique, i.e., harvested when it has reached a height of at least 70–80 cm (not beyond the keg stage). This nutritional strategy involves offering freshly mown sorghum directly in the trough or incorporating it as an ingredient in a total mixed ration (TMR) (Total Mixed Ration) (11, 12). This technique allows to improve milk nutritional characteristics since grass is a profitable source of mineral and vitamins (13). Cut and carry technique has proven to increase productivity in the field, with up to a 25% increase in grass growth rates and a 15% improvement in grass utilization compared to grazing (5). Indeed, by using cut and carry, there is an improvement of land utilization per hectare by 15% and an increase in forage intake (1.6 kg/cow/day) and milk yield, fat and protein compared to grazing (5). Since the latest hybrid fodder varieties contain negligible amounts of dhurrin, the plant can be left to wilt in the field after mowing (only small amounts of hydrocyanic acid are produced), reducing the size of the forage and improving its intake. The hypothesis of this study was that the inclusion of green sorghum in the diet of buffalo cows would have a beneficial effect on the rumen environment, thus improving milk yield and quality. This in turn would have a positive effect on the economic sustainability of buffalo farms. The present trial aimed to evaluate the influence of partially replacing the corn silage with green sorghum in the diet of lactating buffalo cows on milk yield and quality, as well as to estimate the economic sustainability of this feeding system.

## 2 Materials and methods

The study was approved by the Ethical Committee for Animal Care and Use of the University of Naples Federico II (Prot. 2019/0013729 of 8 February 2019) and was conducted from July to September 2024 at a commercial buffalo farm in the province of Caserta (Campania region, Italy, 123 m asl, 41°18′N 14°15′E, with 638 mm mean rainfall and 15.3–25.5 °C mean temperature). In the experimental period (July–September 2024) the temperatures ranged from 20 to 29.2 °C, humidity was 74.6% and 30 mm of rainfall were estimated.

### 2.1 Animals and diet

Forty Italian Mediterranean buffalo cows were equally divided into two groups (C, control and S, treated) homogeneous for parity (3.25 ± 0.4 vs. 3.42 ± 0.3), body weight (BW kg 628 ± 20 vs. 621 ± 27), days in milk (DIM 32.9 ± 6 vs. 36.1 ± 4), previous milk yield (kg 1902 ± 37 vs. 1983 ± 30) and milk yield at the beginning of the study (kg 10.5 ± 2.1 vs. 10.9 ± 2.0). Animals were housed in individual open yards with permanent bedding (8 m^2^/head), exercise area (12 m^2^/head), feeding area (3.5 m^2^/head) and free access to water. The buffalo cows body condition score (BCS) was evaluated weekly on a 1-to-5 scale, where 1 = emaciated, 2 = thin, 3 = average, 4 = fat, and 5 = obese, as suggested by Anitha et al. (14). They were individually fed a total mixed ration (TMR) that differed for the presence of green sorghum (14.7% of TMR on DM basis) harvested at a height of about 1 m, in the diet of group S (Table 1). The experimental period included 20 days of adaptation to diets. The sorghum (*Sorghum vulgare L.*) was a bicolour hybrid x sudanense, early multifloral (Padana Sementi, Tombolo, Padova, Italy), characterised by excellent tillering capacity and very rapid re-sprouting. It is a medium-high plant (2.5–2.6 m), whose highest quality is obtained with harvesting at the keg stage. TMR orts were collected daily to calculate the individual dry matter intake (DMI).

**Table 1:** Ingredients (kg as fed) of total mixed ration for group S and C.

Ingredients	S	C
Corn silage	18.0	20.0
Green sorghum	10.0	–
Alfalfa hay	1.5	2.0
Polyphyte hay*	1.5	2.0
Corn meal	2.0	2.2
Concentrate**	4.7	4.7
Hydrogenated fats	0.1	0.1

*Phleum Pratense L., *Lolium italicum* L., *Trifolium pratense* L.

**Soybean de-hulled grains, cotton meal, sunflower de-hulled grains, wheat bran, dried brewers grains, barley meal, molasses, CaCO3, NaCl, MgO, Sodium bicarbonate, Vit A UI 60000, Vit D3 UI 8000, Vit E mg 90.00, Iodium mg 4.00, MnO mg 200.00, Se mg 0.60, Zn mg 200.00.

### 2.2 Feed analysis

Samples of sorghum and corn silage were collected on a weekly basis. In addition, samples of TMRs were collected before feeding from feed fence at the same intervals. The samples were oven-dried at 65 °C, milled through a 1 mm screen, and analysed for dry matter (DM), crude protein (CP), and ether extract (EE) contents (ID number: 2001.12, 978.04, 920.39 and 978.10, 930.05, respectively), as reported by AOAC (15). The fibre fractions were determined according to Van Soest et al. (16), and starch by the polarimetric method (Polax L, Atago, Tokyo, Japan), as per the official procedure (ISO 6493:2000 28). TMR nutritive value (UFL = 1700 kcal of net energy for lactation) was calculated as suggested by INRA (17). During the last 5 days of the experiment, faecal samples (200 g) were collected four times a day from the rectum of each buffalo cow and analysed as previously reported for the TMR. The *in vivo* digestibility of organic matter (OM), ether extract (EE), crude protein (CP), neutral detergent fiber (NDF) and acid detergent fiber (ADF) was determined using acid insoluble ash (AIA) as an internal marker (18).

### 2.3 Milk analysis

After an adaptation period of 20 days to the diet, the individual milk yield (MY) was recorded daily (by using the TDM software, version 4.1) and averages per animal per month were obtained. Individual milk samples (weighing the two daily milkings) were monthly collected and analysed for protein, fat, lactose and urea by an infrared method using Milko Scan FT 6000 (Foss Matic, Hillerod, Denmark) calibrated for buffalo milk, and somatic cell content (Fossomatic FC^®^ counter, Foss). The mozzarella yield was estimated using the formula suggested by Altiero et al. (19):


Yield%=[3.5(%protein)+1.2(%fat)−0.88]/100.

### 2.4 Statistical analysis

The data from feed analysis were analysed by one-way ANOVA with JMP® software (version 14; SAS Institute. Cary, NC., USA) according to the following model:


yij=μ+Tj+εij.

where: *y* = single data; *μ* = overall mean; *T* = time effect (*j* = 3); *ε* = residual error. For the milk data, two-way ANOVA with JMP software (version 11, PROC GLM, (20)) was used according to the following model:


yijk=μ+Gi+Tj+(GT)ij+d(Gi)+εijk.

where: *y* = single data; *μ* = overall mean; *G* = group effect (*i* = 2; C and S); *T* = time effect (*j* = 3); GT = group × time interaction; *d*(*Gi*) = random effect of buffalo within group; *ε* = residual error. The means were compared using Tukey’s test and the differences considered significant at *p* < 0.05.

## 3 Results

Sorghum showed higher crude protein and NDF contents when compared to corn silage. Nevertheless, the two diets were isonitrogenous and isoenergetic, although the diet of group S had a slightly lower starch content (Table 2).

**Table 2:** Chemical composition (g/kg DM) and nutritive value (UFL/kg DM) of fresh sorghum (FS), corn silage (CS) and TMRs fed by groups C and S.

	FS	CS	TMR C	TMR S
DM %	27.6 ± 1.2	28.1 ± 0.4	55.3 ± 4.8	53.9 ± 5.8
Crude protein	109.5 ± 7.2	87.3 ± 1.8	154.7 ± 14.1	152.2 ± 10.3
Ether extract	30.0 ± 2.1	30.6 ± 0.9	46.7 ± 9.1	46.1 ± 7.4
NDF	581.8 ± 46.3	548.3 ± 36.3	379.5 ± 30.3	388.3 ± 36.2
ADF	324.7 ± 26.4	377. 3 ± 19.9	233.2 ± 20.1	246.1 ± 19.4
ADL	42.1 ± 2.8	49.3 ± 1.3	48.1 ± 5.7	49.3 ± 4.
Ash	95.4 ± 6.2	88.2 ± 6.3	79.1 ± 9.3	78.2 ± 8.3
Starch	68.1 ± 6.8	139.4 ± 6.3	231.3 ± 10.1	204.7 ± 10.5
UFL/kg DM	0.80 ± 0.1	0.80 ± 0.1	0.91 ± 0.2	0.91 ± 0.1

No TMR orts were detected, and the DMI and the BCS were not different between the groups. The OM, CP, EE and starch digestibility did not differ between the groups, while the digestibility (%) of NDF and ADF were significantly (*p* < 0.01) higher in group fed green sorghum (see Table 3).

**Table 3:** Body condition score (BCS), dry matter intake (DMI, kg/day), and nutrient digestibility (%) of groups S and C.

	S	C	*p*-value
BCS	3.2 ± 0.1	3.3 ± 0.4	NS
DMI (kg/day)	16.7 ± 3.1	16.7 ± 2.7	NS
*Digestibility (%)*			
OM	62.3 ± 2.3	58.2 ± 3.0	NS
CP	57.3 ± 1.9	55.1 ± 2.4	NS
EE	85.1 ± 1.1	84.5 ± 1.2	NS
NDF	48.5 ± 2.3	36.8 ± 2.7	<0.01
ADF	42.9 ± 2.7	34.3 ± 3.1	< 0.01
Starch	88.4 ± 1.8	89.3 ± 1.2	NS

NS, not significant; OM, organic matter; CP, crude protein; EE, ether extract; NDF, neutral detergent fiber; ADF, acid detergent fiber.

The mean values of MY, protein and lactose content did not differ between the two groups (Table 4). In contrast, milk fat was significantly (*p* < 0.01) higher in the group fed with green sorghum. In both groups as lactation progressed, there was a significant (*p* < 0.05) decrease in milk production and a simultaneous increase in fat and protein levels. Lactose content, on the other hand, did not significantly vary according to the progress of lactation.

**Table 4:** Milk yield (MY kg/head/d) and chemical composition (g/kg) of groups C and S.

	MY		Fat		Proteins		Lactose	
	C	S	C	S	C	S	C	S
Mean	10.4	10.4	71.2	85.0	43.9	45.0	46.7	46.7
July	11.7	11.4	66.8	85.0	42.9	45.0	46.5	46.6
August	10.8	10.8	69.2	83.0	43.4	45.1	46.9	46.7
September	8.6	8.9	77.6	85.0	45.2	45.0	46.7	47.6
	*p*-value							
Group	NS		**		NS		NS	
Time	*		*		*		NS	
G × T	NS		*		*		NS	
RMSE	6.4		9.1		3.7		2.2	

**p < 0.01; *p < 0.05. NS, not significant; RMSE, root mean square error.

No significant differences were detected for milk somatic cell content; on the contrary, the level of milk urea (MUN) was significantly (*p* < 0.05) lower in group S. Finally, the estimated mozzarella cheese yield (MCY) was higher for the group of buffaloes fed the diet containing green sorghum. It should be noted that this parameter was consistently higher for the S group throughout the trial (Table 5).

**Table 5:** Milk somatic cells (SC, cellules/mL × 1,000), milk urea (MUN, mg/dL) and mozzarella cheese yield (MCY, %) of group C and S.

	SC		MUN		MCY	
	C	S	C	S	C	S
Mean	184.1	217.2	42.4	40.5	23.4	25.3
July	179.3	223.0	40.4	41.1	22.7	25.3
August	159.2	230.1	41.9	41.4	22.9	24.9
September	215.1	200.6	46.4	39.1	24.7	25.6
			*p*-value			
Group	NS		*		*	
Time	NS		*		NS	
G x T	NS		*		NS	
RMSE	89.3		4.2		3.7	

*p < 0.05. NS, not significant; RMSE, root mean square error.

The inclusion of green fodder in the diet could result to be economically advantageous for a lower cost impact and higher estimated mozzarella cheese yield. Table 6.

**Table 6:** Cost (euros/head/day) of rations (kg as fed) for groups S and C.

		S		C	
	€/kg	kg as fed	€	kg as fed	€
CS	0.07	18.0	1.26	20.0	1.40
PH	0.09	1.5	0.14	2.3	0.21
AH	0.15	1.5	0.23	2.0	0.30
CM	0.28	2.0	0.56	2.2	0.62
C	0.4945	4.7	2.32	4.7	2.32
HF	2.0	0.1	0.2	0.1	0.2
GS	0.007	10.0	0.07	–	–
TMR		37.8	4.78	31.3	5.05

CS, corn silage; AH, alfalfa hay; PH, polyphyte hay; CM, corn meal; C, concentrate; HF, hydrogenate fats; GS, green sorghum. The cost of corn silage, alfalfa hay and polyphite hay were determined at the harvesting compared to the local market; that of corn meal was monthly determined using the data collected by the Ager (Borsa Bologna https://www.agerborsamerci.it/listino-borsa/settimanale-ager/) and those of concentrate and hydrogenated fats were monthly collected by the sailing market company.

## 4 Discussion

The present study hypothesized that the inclusion of green fodder sorghum in the diet of buffalo cows would enhance milk yield and composition. This in turn would positively impact the economic sustainability of buffalo farms, due to the increased production and to the reduced input cost.

The chemical composition of green fodder sorghum was in line with that reported in the literature (21). However, the CP content was higher, and NDF content was lower, than the values reported by Uzun et al. (22) who observed 7.1% CP and 45.7%, NDF. This discrepancy may be attributed to differences in the physiological stage at harvest, as Uzun et al. (22) evaluated the forage at the milk stage of development, which, as suggested by Oliveira et al. (23), can substantially influence its nutrient composition. In present study NDF and ADF content falls in the range (500–600 g/kg DM and around 300 g/kg DM, for NDF and ADF, respectively) recommended to avoid decrease in the intake rate and to guarantee the activities of rumen microorganisms and enzymes (24, 25). The replacement of corn silage with green fodder sorghum in the diet did not affect protein and energy concentrations, which were adequate for lactating buffalo cows (26). This result agrees with that reported by Neglia et al. (27) in a trial carried out on buffaloes over 110 days of lactation fed diets characterised by higher protein and energy concentrations (16.5% CP and 0.95 UFL/kg DM). Similarly, no alteration in DMI was recorded and in both groups it was in the range reported by others (28, 29) for lactating buffalo cows confirming the good palatability of green fodder sorghum. As a matter of fact, the buffalo species is known to be wary of the introduction of new feed ingredients in their diet (30, 31). The higher digestibility of NDF and ADF of sorghum compared to corn has been reported in buffalo cows, by Tudisco et al. (6) and in dairy cows by Miron et al. (32). The inclusion of green sorghum in the diet did not lead to a decrease in MY, as also reported by Neglia et al. (27), while milk fat content was significantly higher as also reported by Tudisco et al. (6) on buffaloes fed with sorghum silage. This outcome is probability due to the higher digestibility of the fibrous fractions of diet with sorghum, as confirmed by Calabrò et al. (33) in an *in vitro* trial.

The result concerning milk urea is of particular interest; indeed, the soluble and degradable fractions of diet protein are thought as the main cause of MUN increase (34) because rumen microbes cannot fully capture the excess ammonia (NH₃) when energy support is limited. The surplus NH₃ is therefore absorbed into the bloodstream, converted to urea in the liver, and then excreted in milk and urine. According to Gustafsson et al. (35), fresh forages contain high rumen degradable protein therefore the inclusion of green fodder sorghum in the diet should have result in MUN increase. Instead, Roseler et al. (36) demonstrated that also the contribution of protein by-pass is important in influencing MUN. In fact, these authors fed dairy cows with diets characterized by different ratio of degradable/by-pass protein obtaining similar MUN values. Therefore, other factors (total energy input, fermentable energy input, energy balance fermentable/degradable proteins, rumen nitrogen balance, amino acid composition of the by-pass fraction, lactation stage, productive level) are important as well (37). In the present trial the two diets were isoproteic and isoenergetic; therefore, these two factors would have not be responsible for MUN differences. However, the better utilization of dietary energy by rumen microbes and the increased protein synthesis, due to the higher digestibility of the fibrous fractions, would have led to a decrease in ammonia and therefore urea in the milk. This outcome agreed with the findings of Tajima et al. (38) who reported an increase of rumen cellulolytic bacteria and a decrease of acidotic flora in diet containing fresh forage compared to grain-based diets. Moreover, significantly higher estimated mozzarella cheese yield was recorded in the treated group probably due to the lower MUN, in agreement with the results of Bittante and Cipolat-Gotet (39) who found a significant correlation between high MUN and milk longer rennet coagulation time, weaker curd, and lower mozzarella yield (see Table 7).

**Table 7:** Mozzarella cheese yield (MCY, %) and kg of mozzarella cheese produced by group S and C.

Feed	S	C	S vs. C /day	S vs. C/90 days
MCY (%)	25.3	23.4	1.9	–
Kg Mozzarella cheese	52.6	48.7	+ 3.9	+ 351

Several feeding strategies have already been demonstrated to improve economic sustainability of buffalo farm (40). In the context of the trial, the utilization of sorghum is scheduled during the late spring and summer months, which correspond with the period of higher milk production. The inclusion of green fodder in the diet should resulted to be economically advantageous according to the on-going market trends. The saving obtained was 0.27 EUR/head/day (EUR 5.4/day/group). Therefore, it is estimated that for a mean buffalo farm with 300 buffaloes, the saving would be € 81 per day, which would amount to approximately € 2,500 per month. Furthermore, according to the equation proposed by Altiero et al. (19), the estimated mozzarella yield was greater in group S compared with group C. Moreover, the improved mozzarella yields should also contribute to lowering whey disposal expenses.

Finally, the diet with green sorghum might result in a lower environment impact; indeed, reducing the use of corn silage is supposed to decrease water and energy consumption for irrigation. In addition, Benchaar et al. (41), reported a reduction in methane production by 15 and 21% by using forage with higher digestibility as was the green fodder sorghum in present trial, thus contributing to achieve SDG 13.

## 5 Conclusion

This study provides evidence that the inclusion of green fodder sorghum in the diet of lactating buffalo cows could represent an effective feeding strategy. Its adoption does not adversely affect daily dry matter intake or milk yield, while it significantly enhances milk fat concentration and reduces milk urea content. From an economic standpoint, the use of green fodder sorghum should contribute to a reduction in feeding costs and an improvement in cheese yield, thereby offering tangible economic advantages for producers. Furthermore, the overall environmental impact along the production chain could be mitigated, primarily due to lower irrigation and energy requirements. In conclusion, the inclusion of green sorghum into the diet of lactating buffaloes may constitute a sustainable and economically advantageous approach; nonetheless, further research is warranted to investigate the effects of higher inclusion levels of this feed resource.

## Data Availability

The raw data supporting the conclusions of this article will be made available by the authors, without undue reservation.
